# Regulatory T Cells Modulate CD4 Proliferation after Severe Trauma via IL-10

**DOI:** 10.3390/jcm9041052

**Published:** 2020-04-08

**Authors:** Ramona Sturm, Lara Xanthopoulos, David Heftrig, Elsie Oppermann, Teodora Vrdoljak, Ildiko Rita Dunay, Ingo Marzi, Borna Relja

**Affiliations:** 1 Department of Trauma Surgery, Goethe University, 60590 Frankfurt, Germany; Ramona.Sturm@kgu.de (R.S.); lara_x_@hotmail.de (L.X.); Ingo.Marzi@kgu.de (I.M.); 2 Experimental Radiology, Department of Radiology and Nuclear Medicine, Otto von Guericke University, 39120 Magdeburg, Germany; davidheftrig@googlemail.com; 3 Clinic for Radiology, Neuroradiology and Nuclear Medicine, Klinikum Frankfurt Höchst, 60590 Frankfurt, Germany; 4 Department of General and Visceral Surgery, University Hospital Frankfurt, Goethe-University, 60590 Frankfurt, Germany; Oppermann@em.uni-frankfurt.de; 5 Department of Diagnostic and Interventional Radiology, University Hospital Dubrava, University of Zagreb School of Medicine, 10000 Zagreb, Croatia; Tea.Vrdoljak@gmail.com; 6 Institute of Inflammation and Neurodegeneration, Otto von Guericke University Magdeburg, 39120 Magdeburg, Germany; ildiko.dunay@med.ovgu.de

**Keywords:** proliferation, lymphocytes, regulatory T cells, polytrauma, IL-10

## Abstract

Objective: Severely injured patients frequently develop an immunological imbalance following the traumatic insult, which might result in infectious complications evoked by a persisting immunosuppression. Regulatory T cells (Tregs) maintain the immune homeostasis by suppressing proinflammatory responses, however, their functionality after trauma is unclear. Here, we characterized the role of Tregs in regulating the proliferation of CD4^+^ lymphocytes in traumatized patients (TP). Methods: Peripheral blood was obtained daily from 29 severely injured TP (Injury Severity Score, ISS ≥16) for ten days following admission to the emergency department (ED). Ten healthy volunteers (HV) served as controls. The frequency and activity of Tregs were assessed by flow cytometry. Proliferation of CD4^+^ cells was analyzed either in presence or absence of Tregs, or after blocking of either IL-10 or IL-10R1. Results: The frequencies of CD4^+^CD25^high^ and CD4^+^CD25^+^CD127^−^ Tregs were significantly decreased immediately upon admission of TP to the ED and during the following 10 post-injury days. Compared with HV CD4^+^ T cell proliferation in TP increased significantly upon their admission and on the following days. As expected, CD4^+^CD25^+^CD127^−^ Tregs reduced the proliferation of CD4^+^ cells in HV, nevertheless, CD4^+^ proliferation in TP was increased by Tregs. Neutralization of IL-10 as well as blocking the IL-10R1 increased further CD4^+^ T cell proliferation in Tregs-depleted cultures, thereby confirming an IL-10-mediated mechanism of IL-10-regulated CD4^+^ T cell proliferation. Neutralization of IL-10 in TP decreased CD4^+^ T cell proliferation in Tregs-depleted cultures, whereas blocking of the IL-10R1 receptor had no significant effects. Conclusions: The frequency of Tregs in the CD4^+^ T lymphocyte population is reduced after trauma; however, their inductiveness is increased. The mechanisms of deregulated influence of Tregs on CD4^+^ T cell proliferation are mediated via IL-10 but not via the IL-10R1.

## 1. Introduction

With an incidence of 10%, trauma represents one of the most common causes of death worldwide, and it remains the leading cause of death in young patients (<40 years of age) [1,2]. The most frequent causes of post-traumatic mortality which occurs within the first 72 h after the insult are caused by severe injuries to the central nervous system and/or hemorrhagic shock due to a significant blood loss. On the contrary, late post-injury mortality is often caused by secondary complications including sepsis, acute respiratory distress syndrome, and/or multiple organ dysfunction syndrome [3,4,5]. In the past, significant improvements in pre-clinical and clinical treatment strategies slightly reduced the post-traumatic mortality, particularly by addressing hemorrhagic shock, however, the rate of multiple organ failure induced by infectious complications has increased [5,6].

The multifactorial biological response to tissue injury after severe trauma triggers an uncontrolled inflammatory reaction. Endogenous damage-associated molecular patterns as well as pathogen-associated molecular patterns activate effector cells of the innate immunity [7,8,9,10,11,12,13]. In parallel, a counterbalancing anti-inflammatory response syndrome (CARS) can develop [12,14]. Thus, traumatized patients remain at risk for the development of a persistent inflammation, immunosuppression and catabolism syndrome (PICS) [15]. Neither cellular nor humoral mechanisms of the post-traumatic immunosuppression are fully understood. Depending on the prevailing cytokine milieu T cells develop either into the proinflammatory TH1 or the anti-inflammatory TH2 cell type. IL-10 and its inhibitory potency have been associated with a TH1/TH2 shift, which seems to be mediated by regulatory T cells (Tregs) [16,17,18,19]. In 1995, Sakaguchi et al. identified a subpopulation of CD4^+^ T cells in mice that continuously expressed the α-chain of the IL-2 receptor (CD25) [20]. These Tregs can also be defined by the absence of the α-chain of the IL-7 receptor (CD127) [21,22] and by the expression of the transcription factor Forkhead box (Fox)P3 [23]. In the field of trauma research, different combinations mainly of the cellular surface markers have been applied to detect Tregs, however, a unified definition of Tregs according to their antigen expression has not been found yet [24,25]. With regard to their functions, it is known that Tregs can modulate and kill antigen presenting cells as well as effector T cells, and thus, that they may affect both innate and adaptive immunity [26]. It was demonstrated that the activity and percentage of Tregs were increased after trauma, but that their absolute numbers were reduced [25,27]. Interestingly, it was shown that anti-inflammatory cytokine IL-10 increased significantly in septic patients [28,29,30]. Stratifying major surgical patients to those who develop sepsis versus those without complications has uncovered that the last had significantly reduced IL-10 levels [31]. Aligned with these findings, IL-10 levels were elevated in traumatized patients with complications compared to those without complication [32]. Taken together, previous reports suggest that IL-10 is critically involved in immunosuppression following trauma, and further, that it is associated with the occurrence of post-traumatic complications. While the production of IL-10 by CD4^+^CD25^+^ cells was significantly reduced, the IL-10 production by Tregs was increased after trauma [24]. Thus, it appears reasonable that both IL-10 and Tregs play significant roles in the onset of post-traumatic immunosuppression. Yet, whether the inhibition of T cell-mediated inflammation is potentially regulated by Tregs or IL-10 itself deriving from other cells still remains unclear. However, to prevent infectious complications in traumatized patients, it is crucial to understand the mechanistical background of the post-traumatic immunosuppression.

## 2. Experimental Section

### 2.1. Ethics

This study was performed in the University Hospital of the Goethe-University Frankfurt with institutional ethics committee approval (312/10) in accordance with the Declaration of Helsinki and following the Strengthening the Reporting of Observational Studies in Epidemiology guidelines [33]. All enrolled patients or their legally authorized representative and all healthy volunteers signed the written informed consent form.

### 2.2. Study Setting and Population

Twenty-nine severely injured traumatized patients (TP) between 18 and 80 years of age with an Injury Severity Score (ISS) ≥16 were enrolled into this study immediately at admittance to the emergency department (ED). Inclusion criteria covered acute blunt or penetrating trauma. Exclusion criteria were pre-existing immunological disorders, HIV, infectious hepatitis, immune-suppressive medication, acute myocardial infarction, burns and/or thromboembolic events. Ten healthy volunteers served as controls. 

### 2.3. Study Protocol

At admittance to the ED all TP were treated according to the polytrauma guidelines. Vital parameters were recorded. The ISS was determined based on the Abbreviated Injury Scale [34,35] of the six body regions. The sum of the squares of the three most severely injured body regions was applied to generate the overall injury severity (ISS). 

### 2.4. Blood Sampling

Blood samples were taken as early as possible after admittance of the patient to the ED and daily for the following ten days between 7 and 11 a.m. Blood was withdrawn in ethylenediaminetetraacetic acid (EDTA) tubes (Sarstedt, Nürmbrecht, Germany) and kept either at room temperature for functional assays or on ice for flow cytometric analyses.

### 2.5. Analyses of Cell Surface Receptors

**Figure 1:**
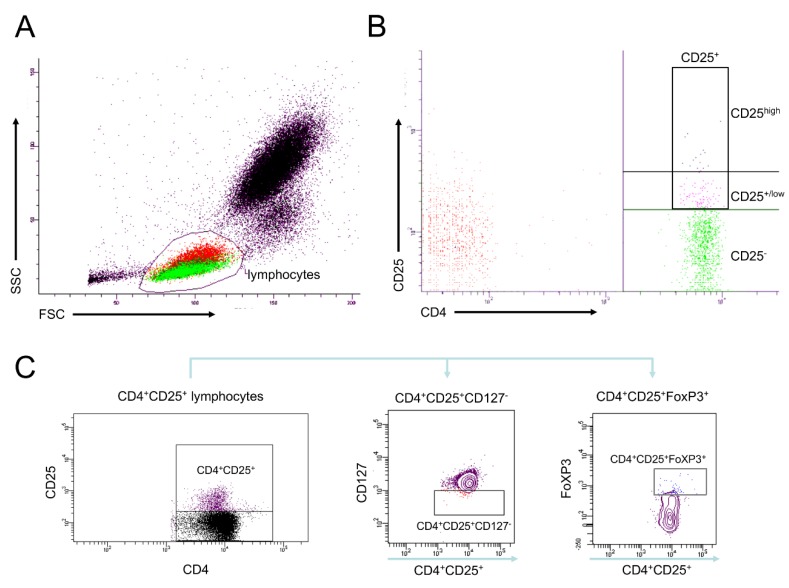
Gating strategy for the flow cytometric analysis. (**A**): Gating of the lymphocyte fraction in peripheral blood according to the forward and side scatter. (**B**): Gating for the detection of CD4^+^CD25^+^ cells. (**C**): Gating of the lymphocytes according to their CD4 and CD25 expression and subsequently of CD127 and FoxP3 expressing cells.

After the blood samples (100 µl) were transferred into polystyrene Falcon tubes (BD PharMingen, San Diego, CA, USA), they were diluted with 395 µl RPMI 1640 (Gibco) and finally 5 µl of the leukocyte activation cocktail (LAC, BD PharMingen, San Diego, CA, USA) were added. The samples were incubated for 5 h at 37 °C and 5% CO_2_. Corresponding samples without stimulation served as controls. Centrifugation continued at 400× *g* for 5 min and the supernatants were removed. One hundred microliters of the Fix & Perm Solution A (Fix & Perm (R) ADG-GAS-002) for the intracellular staining were added. After 15 min at room temperature, cells were washed with phosphate-buffered saline (PBS), and centrifugation step followed at 400× *g* for 5 min. The supernatants were removed and 100 µl of the Fix & Perm Solution B were added. The samples were incubated for 30 min at room temperature in the dark with mouse anti-human CD4 Brilliant Violett (Clone RPA-T4, BD Bioscience, San Diego, CA, USA), mouse anti-human CD127 PerCP-Cy5.5 (Clone HIL-7R-M21, BD Bioscience, San Diego, CA, USA), mouse anti-human CD25 APC-Cy7 (Clone BC 96, BD Bioscience, San Diego, CA, USA), and mouse anti-human FoxP3 PE-Cy7 (Clone PCH101, BD Bioscience, San Diego, CA, USA) antibodies. Subsequently, 2 mL of the FACS lysing solution (BD PharMingen, San Diego, CA, USA) were added for additional 10 min. Then samples were washed twice with FACS buffer (PBS supplemented with 0.5% bovine serum albumin, BSA). Supernatants were removed, cells were diluted in 300 µl FACS buffer and immediately subjected to flow cytometric analyses with a BD FACS Canto II using a FACS DIVA software (BD, San Diego, CA, USA). The lymphocytes were defined by gating CD4^+^ cells in the corresponding forward and side scatter scan. Positively gated cells were determined by their absolute cell numbers and the percentage of the selected parietal cell population were measured. Gating strategy is shown in Figure 1. 

### 2.6. Isolation of CD4^+^ Cells Including Tregs

CD4^+^ leukocytes were isolated using Ficoll density gradient centrifugation (Ficoll solution, 1.077 g/mL; Biochrom GmbH, Berlin, Germany) at 800× *g* for 20 min at room temperature without break. Then, the mononuclear cell layer in the interface was removed and cells were washed twice with MACS buffer (PBS + BSA 0.5% + EDTA 2 mM). Leukocytes were isolated by negative selection using a Biotin-Antibody-Cocktail including CD8a, CD14, CD15, CD16, CD19, CD36, CD56, CD132, TcRγ/δ, and CD235a (CD4 T-cell Isolation Kit, Miltenyi Biotec, Bergisch Gladbach, Germany) according to the manufacturer’s instructions. Also, CD71 and CD8 were removed by using CD71 and CD8 MicroBeads (Miltenyi Biotec Bergisch Gladbach, Germany). Subsequently, cells were applied for the proliferation assay of CD4^+^ cells including Tregs.

### 2.7. Isolation of CD4^+^ Cells without Tregs

After their isolation, CD4^+^ cells were incubated with CD127 MicroBeads (Miltenyi Biotec, Bergisch Gladbach, Germany) for negative selection. CD4^+^CD127^+^ cells were removed from the column and placed on ice. Briefly, Tregs were isolated by incubating the CD4^+^CD127^−^ cells with CD25 MicroBeads (Miltenyi Biotec, Bergisch Gladbach, Germany) by positive selection. The flow through with CD4^+^CD127^−^CD25^−^ cells constituted with pre-gained CD4^+^CD127^+^ cells the CD4^+^ leukocyte population without Tregs.

### 2.8. Proliferation of Lymphocytes in a CD4^+^ Culture with and without Tregs

Following their isolation, CD4^+^ T cells with Tregs as well as CD4^+^ T cells without Tregs were immediately used for experiments. 50,000 cells were diluted in 200 µl RPMI-1640 (with supplements of penicillin-streptomycin, gentamycinsulfate, and heat inactivated FBS) and seeded in 96-well plates (BD Biosciences, Franklin Lakes, NJ, USA). Previously, the wells were coated with anti-CD3 (2 μg/mL, BD PharMingen, Heidelberg, Germany) for 24 h at 4 °C. For co-stimulation of T cells, anti-CD28 (100 μg/mL, BD PharMingen, San Diego, CA, USA) was applied at the experimental day. IgG1κ Isotype Control Purified and NA/LE Mouse 107.3 were used as corresponding isotype controls (BD PharMingen, San Diego, CA, USA). For IL-10 neutralization or IL-10 receptor blocking, anti-IL-10 (JES3-19F1) or anti-IL10-receptor (3F9, 500 μg/mL, BD Bioscience, San Diego, CA, USA) antibodies were added. IgG2a Isotype Control Purified NA/LE Rat R35-95 antibody was used as control (BD PharMingen, San Diego, CA, USA). After 24 h at 37 °C and 5% CO_2_ 100 µl of the culture medium were replaced and either the anti-IL-10 or the anti-IL10-receptor antibody were added again. After 48 h supernatants were removed and cells were treated with the BrdU labeling solution (Cell Proliferation ELISA BrdU, La Roche, Basel, Switzerland) according to the manufacturer’s instructions. The proliferation rates were detected by measuring the colorimetry using a Tecan microplate reader and Magellan software.

### 2.9. Statistical Analysis

GraphPad Prism 6.0 software (GraphPad Software Inc. San Diego, CA, USA) was used to perform the statistical analysis. Data are given as mean ± standard error of the mean (SEM) or as absolute cell numbers calculated in percent. A Student’s *t*-test with Welch correction and one-way analysis of variance (ANOVA) with a Dunn post-hoc test was used for comparisons among the groups. A *p* value below 0.05 was considered statistically significant. 

## 3. Results

### 3.1. Main Findings

#### 3.1.1. Study Population

Twenty-nine patients with severe trauma (TP) and 10 healthy volunteers (HV) were enrolled in this study. The mean age of the patients was 44.6 ± 2.3 versus 37.6 ± 1.7 years of age. Seventy-five percent of patients were male. All patients were substantially injured with an ISS of 30.3 ± 2.3. The mean stay in the intensive care unit was 9.7 ± 2.6 days, and the total duration of the in-hospital stay was 19.3 ± 3.4 days. The data is comparable to mean values of the multiply injured trauma patients as demonstrated before [36,37].

#### 3.1.2. Frequencies of Differentially Defined Tregs after Trauma

**Figure 2:**
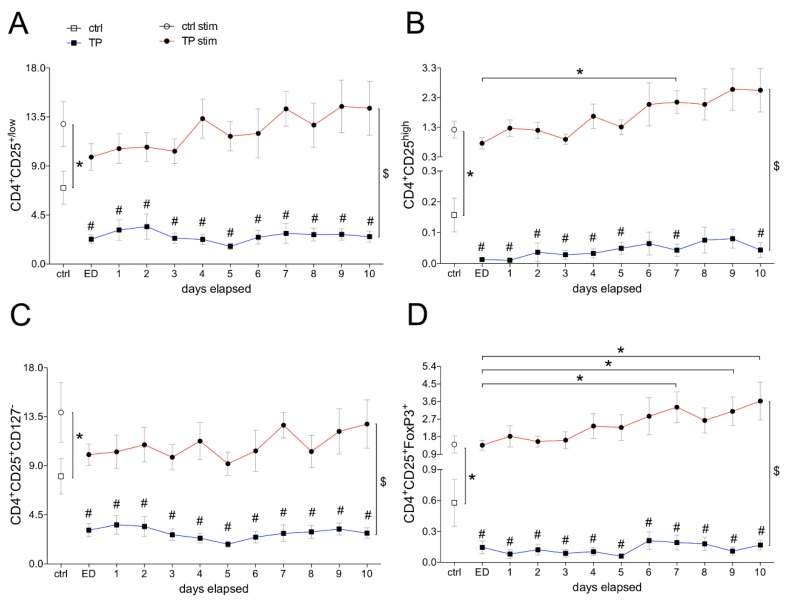
Whole blood from healthy volunteers (ctrl, *n* = 10, clear symbols) or major trauma patients (TP, *n* = 29, black symbols) was analyzed by flow cytometry over a 10-day time course after admission (emergency department, ED-10). Regulatory T cells (Tregs) were detected using anti-human CD4 and CD25 in the corresponding side and forward scatter. Unstimulated (square) and stimulated (circle, stim) cells were measured. Data are shown as mean ± SEM percentage of lymphocytes. (**A**): CD4^+^CD25^+/low^ Tregs to CD4^+^ lymphocytes; (**B**): CD4^+^CD25^+/high^ Tregs to CD4^+^ lymphocytes; (**C**): CD4^+^CD25^+^CD127^−^ Tregs to CD4^+^ lymphocytes; (**D**): CD4^+^CD25^+^FoxP3^+^ Tregs to CD4^+^ lymphocytes. *p <* 0.05 * vs. indicated #; unstimulated TP vs. unstimulated crtl $; stimulated TP vs. unstimulated TP.

Of all CD4^+^ lymphocytes from HV, 6.9% had low CD25 expression. TPs had significantly lower percentage of CD4^+^CD25^+/low^ lymphocytes in total leukocyte population at admission to the ED compared to ctrl (*p <* 0.05, Figure 2A), and this difference persisted during the complete observational time course (Figure 2A). LAC stimulation increased significantly the presence of CD4^+^CD25^+/low^ in HV to 12.9% compared to unstimulated samples from ctrl (*p <* 0.05). The low presence of CD4^+^CD25^+/low^ cells in TP was significantly enhanced after LAC stimulation compared to unstimulated samples from TP during the complete observational course (*p <* 0.05). Upon stimulation, the presence of CD4^+^CD25^+/low^ increased significantly compared to unstimulated samples over the ten days course and remained in the range of stimulated samples from HV (Figure 2A).

The proportion of CD4^+^CD25^+/high^ cells was significantly reduced compared to ctrl immediately upon admission to the ED and remained diminished during the complete observational period (Figure 2B). In vitro stimulation increased significantly the proportion of CD4^+^CD25^+/high^ cells compared with unstimulated samples in both groups. Upon stimulation at post-injury day 7, the presence of CD4^+^CD25^+/high^ cells in TP was significantly enhanced compared to stimulated samples from ctrl (Figure 2B).

During the whole observational period, the ratio of CD4^+^CD25^+^CD127^−^ cells to total CD4 lymphocytes remained significantly lower in TP compared to ctrl (*p <* 0.05, Figure 2C). After the in vitro stimulation with LAC, CD4^+^CD25^+^CD127^−^ cells increased significantly in both TP and ctrl compared to the corresponding unstimulated samples (*p <* 0.05, Figure 2C).

During the whole observational period of ten days, the ratio of CD4^+^CD25^+^FoxP3^+^ cells to total CD4 lymphocytes remained significantly lower in TP compared to ctrl (*p <* 0.05, Figure 2D). After the in vitro stimulation with LAC, CD4^+^CD25^+^FoxP3^+^ cells increased significantly in both TP and ctrl compared to the corresponding unstimulated samples (*p <* 0.05, Figure 2D). Upon stimulation at post-injury days 7, 9, and 10, the presence of CD4^+^CD25^+^FoxP3^+^ cells in TP was significantly enhanced compared to stimulated samples from ctrl (*p <* 0.05, Figure 2D).

#### 3.1.3. Proliferation of CD4^+^ Cells Depending on Tregs

**Figure 3:**
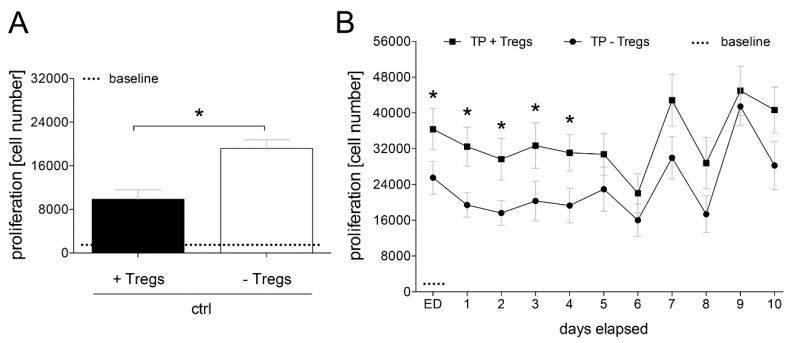
CD4^+^ T lymphocytes were isolated from healthy volunteers (ctrl) or major trauma patients (TP) and depleted from regulatory T cells (Tregs) over a 10-day time course after admission (emergency department, ED-10). (**A**): The proliferation rates in absolute cell numbers of CD4^+^ cells with (+) and without (−) Tregs in healthy volunteers and in (**B**): severely traumatized patients is shown. Data are shown as mean ± SEM. *p <* 0.05 *; +Tregs vs. −Tregs.

Depletion of Tregs out of the CD4^+^ lymphocyte population significantly increased the CD4^+^ T cell proliferation compared to CD4^+^ cells which still contained the Tregs fraction (*p <* 0.05, Figure 3A).

The proliferation rate of CD4^+^ cells containing the Tregs fraction was significantly enhanced in TP during the whole observational period compared to ctrl (*p <* 0.05). Immediately at admission of the TP to the ED as well as during the first four post-injury days, depletion of Tregs out of the CD4^+^ lymphocyte population significantly decreased the CD4^+^ T cell proliferation compared to CD4^+^ with Tregs (*p <* 0.05, Figure 3B).

#### 3.1.4. Tregs-Mediated Proliferation of CD4^+^ Cells Depends on IL-10

**Figure 4:**
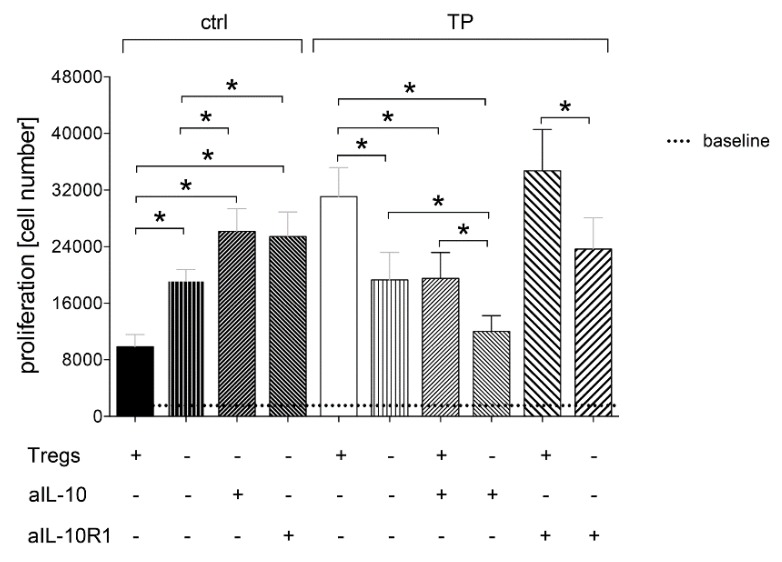
The proliferation rates in absolute cell numbers of CD4^+^ cells with (+) and without (−) regulatory T cells (Tregs) in healthy volunteers (ctrl, *n* = 10) and severely traumatized patients (TP, *n* = 25) as well as depending on IL-10 neutralization (aIL-10) and IL-10 receptor blockade (aIL-10R1) is shown. Data are shown as mean ± SEM. *p <* 0.05 * vs. indicated.

The proliferation rate of CD4^+^ lymphocytes depleted from Tregs was significantly increased compared to the co-culture of CD4^+^ cells and Tregs in HV ctrl (*p <* 0.05, Figure 4). The proliferation rate of CD4^+^ lymphocytes depleted from Tregs was significantly decreased compared to the co-culture of CD4^+^ cells and Tregs in TP at admission to the ED (*p <* 0.05, Figure 4).

To determine the infuence of IL-10 on the Tregs regulated proliferative behavior of CD4^+^ cells, either IL-10 was neutralized or the IL-10R1 has been blocked. In the ctrl group, both neutralization of IL-10 as well as blocking the IL-10R1 in Tregs-depleted CD4^+^ cultures increased further and significantly the proliferation rates compared to the proliferation rate of the Tregs depleted CD4^+^ culture (*p <* 0.05). No significant differences in proliferation were observed between the IL-10-neutralized or IL-10R1-blocked Tregs-depleted CD4^+^ cells in ctrl (Figure 4). Furthermore, the control experiments with CD4^+^ cells containing Tregs population and undergoing both IL-10 as well as IL-10R1 neutralization show that Tregs apparently suppress the T cell proliferation by direct cell contacts, since the results were comparable to proliferation rates from the untreated controls without Tregs depletion. However, considering the above described results, both IL-10 or IL-10R1 do play a suppressive role in T cell proliferation under control conditions. 

Significantly increased proliferation rate of CD4^+^ lymphocytes including Tregs versus Tregs-depleted CD4^+^ cells in TP was significantly decreased upon the neutralization of IL-10 (*p <* 0.05, Figure 4). There was no difference in the proliferation rate between Tregs-containing CD4^+^ cell culture with IL-10 neutralization and Tregs-depleted CD4^+^ cell culture without IL-10 neutralization in TP. Nevertheless, the proliferation rate in Tregs-depleted CD4^+^ cell culture with IL-10 neutralization was significantly reduced compared to Tregs-depleted CD4^+^ cell culture in TP at ED (*p <* 0.05, Figure 4). The data indicate that IL-10 can reduce the CD4^+^ lymphocyte proliferation irrespective of the presence of Tregs in the cell culture in TP at ED. 

Significantly increased proliferation rate of CD4^+^ lymphocytes including Tregs versus Tregs-depleted CD4^+^ cells in TP was not changed upon blocking of the IL-10R1 in TP at ED (Figure 4). These data indicate that IL-10R1 does not mediate the effects on the CD4^+^ lymphocyte proliferation in TP at ED which were induced by Tregs. 

Briefly, the proliferation rate of Tregs-depleted CD4^+^ lymphocytes was statistically comparable between ctrl and TP (Figure 4). However, while the neutralization of IL-10 in Tregs-depleted CD4^+^ lymphocytes significantly increased the proliferation rate in ctrl, the same approach significantly reduced the proliferation of Tregs-depleted CD4^+^ lymphocytes in TP at ED (*p <* 0.05, Figure 4).

While blocking of the IL-10R1 in Tregs-depleted CD4^+^ lymphocytes significantly increased the proliferation rate in ctrl (*p <* 0.05), the same experimental approach resulted in comparable results in cells which were obtained from TP at ED, although the last was not significant (Figure 4).

## 4. Discussion

Aim of the present study was to phenotype Tregs after polytrauma and to determine their ratio in the cellular fraction of CD4^+^ lymphocytes over an observational period of ten post-injury days. In addition, the underlying mechanism of Tregs-modulated proliferation of T cells via IL-10 or the IL-10 receptor, respectively, were elaborated after polytrauma. 

Particularly the functions of CD4^+^ T cells, which are essential for an effective defense against invading pathogens after trauma are reduced during CARS or PICS [15]. Thus, this immunological imbalance might result in serious complications, sepsis and (multiple) organ failure in the later post-injury phase. T cell suppression was associated with an increased rate of complications as well as enhanced mortality rates after severe injury [32]. Interestingly, an increased number of Tregs, which can inhibit T cell proliferation and their functions via IL-10 as well as increased IL-10 levels were associated with the development of post-traumatic complications and mortality [25,38,39,40]. A reduced number of Tregs with their increased activity after trauma were reported [27]. Previous studies report an increased proportion of Tregs in the CD4^+^ T cell population after polytrauma [24]. Still, there is a lack of continuous follow-up studies regarding the frequency of Tregs in the CD4^+^ T cell population and a comparison of the differentially and still inconsistently defined Tregs populations after polytrauma. Besides, it is also unclear whether Tregs exerts their inhibiting effects on the proliferative capacity of CD4^+^ lymphocytes via IL-10 after severe injury. There are suggestions that Tregs inhibit T cell proliferation via TGF-β and IL-10 [39,41]. However, other studies propose that Tregs inhibit T cell proliferation via direct cell contacts independently of IL-10 [42]. Our control experiments also show that Tregs apparently suppress the T cell proliferation by direct cell contacts as already demonstrated by Levings et al. [42]. Although the authors suggest that Tregs inhibit T cell proliferation via direct cell contacts independently of IL-10, in our study both IL-10 or IL-10R1 do play a suppressive role in T cell proliferation under control conditions. Despite the depletion of Tregs, neutralization of both IL-10 as well as IL-10R1 further increased T cell proliferation. On the contrary, the suppressive activity of Tregs on T cell proliferation might not be triggered by direct cell contacts. Under traumatic conditions depletion of Tregs reduced T cell proliferation, which is a paradox in their natural and characteristic functions. In line with this, the presence of Tregs increased T cell proliferation. Blocking IL-10 in presence of Tregs reduced proliferation, though a depletion of Tregs with IL-10 blocking further decreased T cell proliferation. In summary, the direct proliferation-suppression which is observed under control conditions in healthy volunteers appears to be deregulated in trauma, and in this setting, IL-10 may be involved in the changed functionality of Tregs. Since IL-10R1 blockade under traumatic conditions has provided comparable results to healthy volunteers, the data suggest that the receptor exerts its functions. Thus, the results propose that the cell contact dependent suppression of T cell proliferation via Tregs may be closely linked to IL-10, and furthermore, that it may be associated with its unknown functions under traumatic conditions. Thus, the impact of Tregs on the T cell proliferation in polytraumatized patients and potential role of IL-10 still remain unknown.

MacConmara et al. examined the distribution and activation markers of Tregs in the lymphocyte population using peripheral blood from 19 polytraumatized patients (ISS: 36.6 ± 13.9) within 24 h after admission to the emergency department and after 7 days [24]. In contrast to the results of our study, the proportion of CD25-expressing cells immediately after trauma was comparable to healthy volunteers. At day 7, the proportion of CD25^+^ cells and CD25^+/high^ cells increased significantly, data that were not confirmed by our study since we did not observe an increase in the CD25^+^ population. On the contrary, this specific population was reduced immediately at admission and during the ten post-injury days. However, the ex vivo in vitro stimulation induced Tregs. In the paper by MacConmara et al., the higher injury severity of their patients with enhanced complication rates may have induced these cells. 

Hein et al. demonstrated that the absolute number of CD4^+^CD25^+^CD127^-^ Tregs decreased immediately after a septic shock, but their proportion out of CD4^+^ cells remained the same compared to healthy volunteers [25]. After 3 days, however, both the proportion and their absolute number increased. One week later, the absolute number of Tregs was comparable to healthy volunteers, but their percentage remained elevated due to the severe lymphopenia in septic patients [25]. In our study, there was a significant decrease of CD4^+^CD25^+^CD127^−^ and CD4^+^CD25^+/high^ Tregs already at admission to the emergency department which persisted for the following 10 days. The data from Hein et al. demonstrated that lymphopenia was more pronounced in septic patients compared to non-septic patients, which actually have been mainly analyzed in our study. This might explain why the percentage of Tregs in our work was lower. Thus, based on the current literature, Tregs do have an important but not yet profoundly characterized role during the post-traumatic inflammation. They may be an important determinant of both extent and severity of the post-traumatic immunosuppression [18,43]. Since most patients dying from severe clinical problems including sepsis show signs of immunosuppression [44], there are reasonable suggestions that Tregs play an important role in the development of infectious complications after trauma. It has been demonstrated that both their number and functions increase following the onset of severe sepsis or septic shock [43]. In burn trauma models it has been shown that severe burn injury per se could change Tregs activities. As demonstrated by increased levels of cytokines produced by those cells and their activation markers, they might play an important role in the pathogenesis of sepsis as well as mortality in patients suffering from burn injuries [45]. Recently, Kramer et al. have demonstrated that the depletion of Tregs attenuated T cell brain infiltration, reactive astrogliosis, interferon-γ gene expression, and transiently motor deficits in murine acute traumatic brain injury [46]. Thus, the current literature does suggest that regulatory T cells contribute to post-traumatic complications, however, in our study only two patients during their stay in the intensive care unit have been diagnosed with sepsis. It is not possible to perform any statistical evaluations based on two patients, and therefore, further studies with a larger number of septic patients after trauma are required to clarify the importance of Tregs in post-traumatic sepsis.

Interestingly, our results are comparable to data obtained from a trauma model in rats consisting of a bilateral femoral fracture and hemorrhagic shock [47]. Four hours after trauma induction, the number of CD4^+^CD25^+/high^FoxP3^+^ Tregs in peripheral blood was significantly lower compared to controls. In addition, the number of Tregs correlated negatively with the histological extent of injury severity [47].

When interpreting our data regarding the cell phenotype, we must indicate the application of LAC with Phorbol 12-Myristate 13-Acetate (PMA) and a calcium ionophore Ionomycin (PMA/Iono) for stimulation, which is commonly used for the detection of intracellular accumulations of cytokines by intracellular staining. Although the applied PMA/Iono stimulation is mainly utilized to elicit a primary cytokine response or intracellular proteins from T cells, its use does also activate Tregs. In line, Wang et al. have shown that regulatory T cells of the CD4^+^CD25^+^FoxP3^+^CD127^−^ phenotype were inducible by PMA/Iono stimulation [48]. This phenotype is comparable to those that have been detected in our study. Although CD3CD4 expression was suppressed by PMA/Iono, the proportion of CD4CD25FoxP3CD127 increased after PMA/Iono stimulation as shown by Wang et al. [48]. In addition, this data demonstrates that polyclonal stimulation using PMA/Iono can induce CD4CD25FoxP3IL-2 Treg in vitro. Another study described four phenotypically determined regulatory T cell phenotypes in peripheral blood lymphocytes, which were commonly characterized as CD4^+^CD25^high^, CD4^+^CD25^high^FoxP3^+^, CD4^+^CD25^high^CD127^−^, or CD4^+^CD25^high^FoxP3^+^CD127^−^ cells were increased upon PMA/Iono stimulation [49]. Importantly, PMA/Iono can also reduce antigen surface expression and specifically CD4 expression as demonstrated by Wang et al. and others [50,51,52]. Importantly, it has also been shown that the proportion of FoxP3^+^CD127^-^ peripheral blood lymphocytes increased with the quantity of CD25 on stimulated CD4^+^ peripheral blood lymphocytes, and was highest in CD25^high^ peripheral blood lymphocytes, emphasizing the relevance of CD25^high^ as regulatory T cell marker [49]. Briefly, the data show that although CD3CD4 expression is suppressed by PMA/Iono, the proportion of CD4CD25FoxP3CD127 increases after PMA/Iono stimulation. These data as well as considering that the ratio of regulatory T cells to lymphocytes specifically expressing CD4 was evaluated in our study, the approach is reasonable. However, PMA/Iono as the standard polyclonal stimulus for T cell and inducible Treg [53] down-regulating CD4 expression quickly and severely must be considered as an important disadvantage for certain studies requiring separation of regulatory T cells. This was considered in our methodological settings, and therefore, Tregs have been separated from the T cell population before any stimuli were applied. 

A post-traumatic anergy of CD4^+^ T cells with their reduced numbers and activity has been described. Interestingly, despite CD3/CD28 stimulation, T cells isolated from severely injured patients with multiple organ dysfunction syndromes were not able to proliferate post-traumatically. In the present study, however, a significant post-traumatic increase in CD4^+^ T cell proliferation was observed. Only by depleting Tregs from the CD4^+^ T cell population reduced their proliferative capacity after trauma. With regard to the post-traumatic CD4^+^ T cells anergy, it must be considered that no distinction was made between different T cell subgroups, and thus, only certain CD4^+^ T cell subsets may have shown post-traumatically altered proliferation. 

Beacher-Allan et al. have shown that the suppressive properties of CD4^+^CD25^+/high^ Tregs and the suppressed proliferation depended on the concentration of the applied stimulant [38]. In summary, Tregs have a contrary effect on T cell proliferation in trauma patients compared to healthy volunteers. This effect may be caused by an altered functionality of Tregs or by an altered effect of Tregs-associated cytokines on CD4^+^ T cells. IL-10 mediated inhibition of T cells mainly leads to the inhibition of TH1 cells and their pro-inflammatory cytokines such as IFN-γ [54]. Waal Malefyt et al. demonstrated that although T cell proliferation was stimulated by monocytes, IL-10 significantly inhibited the proliferation depending on its concentration [55]. Our results show that the inhibitory effect of Tregs on T cell proliferation can be eliminated by IL-10 neutralization in healthy volunteers. Taken together, the data suggests that Tregs may exert their inhibitory effects on T cell proliferation via IL-10, contradicting the assumption that Tregs act independently of IL-10. In trauma patients, increased IL-10 levels were associated with enhanced complication rates of sepsis, CARS and increased mortality [28,29]. Remarkably, our data indicate that IL-10 exerted proliferation-increasing effects, since its neutralization reduced proliferation rates after trauma. This intriguing observation in Tregs depleted CD4^+^ cultures may have been caused by other proliferation-inhibiting CD4^+^ T cell types that potentially produced IL-10. Our results suggest that Tregs inhibit T cell proliferation via IL-10. However, IL-10 mainly affects TH1 cells rather than TH2 and in trauma there are reports suggesting a shift of TH1 towards TH2 cells. This may explain why Tregs cannot affect T cell proliferation after trauma to the same extent as in healthy individuals.

With regard to the functions of IL-10 receptor in polytraumatized patients, there are only sparse data in the literature. Wang et al. described that the continuous IL-10R blockade in spleen cells of infected mice significantly increased the IL-10 concentration compared to healthy mice [56]. In addition, IL-10R blockade increased the secretion of pro-inflammatory cytokines, reduced the survival time as well as the amount and efficacy of Tregs [56]. However, we did not observe any significant differences in proliferative capacity after receptor blockade. The proliferation-increasing effect induced by Tregs after trauma was detectable despite blocking the IL-10 receptor. Thus, blocking the IL-10 receptor does not result in the same effects as IL-10 neutralization in traumatized patients. To define the exact role of IL-10 and its receptor in Tregs after polytrauma further in vivo studies will be required.

Limitations of this study certainly include the confined number of traumatized patients that have been involved. In addition, only two patients developed septic complications during their stay in the intensive care unit and two patients died. This kind of study utilizes medical personnel as well as costs to a great extent, since the development of post-traumatic complications is not predictable, and thus all patients must be included prospectively in the analyses before they develop complications. Thus, clarifying the specific role of Tregs as well as their altered functions in sepsis development or mortality after polytrauma must be elaborated in larger studies in future. Also, further in vivo studies to evaluate the mechanistical and pathophysiologal role of IL-10 as well as assessing of potential therapeutic targeting strategies in this setting are necessary. Besides, with regard to the definition of Tregs in general, so far, no consensus was reached on the use of a specific set of markers when identifying Tregs in human and mice. We have chosen CD25 to stratify the different phenotypes and their dynamics, yet, it remains to be noted that we did not evaluate the direct immunosuppressive activity and functionality of different phenotypes, and that this remains to be elucidated in further studies as well. 

Taken together, both regulatory T cells as well as IL-10 have different effects on CD4^+^ T cell proliferation after trauma compared to healthy controls. Here, Tregs-induced CD4^+^ T cell proliferation after trauma may be mediated by IL-10. On the contrary, IL-10 receptor does not appear to be directly involved in those effects of Tregs after trauma. The potential involvement of another receptor for IL-10 remains to be elaborated in further studies.
